# The effects of French contrast training on lower limb athletic performance in healthy adults: a systematic review and meta-analysis

**DOI:** 10.3389/fphys.2025.1672353

**Published:** 2025-08-21

**Authors:** Ziren Zhao, Zhili Ma, Chao Wu, Xin Zheng, Tingyuan Liu, Nijiao Deng, Kaixiang Zhou

**Affiliations:** ^1^ College of Physical Education and Health Science, Chongqing Normal University, Chongqing, China; ^2^ College of Sports and Health, Chengdu University of Traditional Chinese Medicine, Chengdu, Sichuan, China; ^3^ Physical Education Department, University of International Business and Economics, Beijing, China

**Keywords:** French contrast training, athletic performance, jump, sprint, maximal strength

## Abstract

**Background:**

French Contrast Training (FCT) is a unique complex training (CT) method that has gained attention in sports science. Participant characteristics, training protocols, and control group types may influence the effects of FCT on lower limb athletic performance.

**Objective:**

The aim of this systematic review and meta-analysis was to assess the effects of FCT on jump, sprint, and maximal strength in healthy adults and to identify potential moderators of training effects.

**Methods:**

We searched five databases (PubMed, Web of Science, Embase, EBSCO, and CNKI). Seven randomized controlled trials with 195 participants were included. We used a random-effects model for the outcomes (jump, sprint, and maximal strength) to calculate the pooled effect sizes (standardized mean differences, SMD). Subgroup analyses and GRADE assessments were conducted to explore heterogeneity and assess the quality of evidence.

**Results:**

FCT significantly improved sprint (large effect: SMD = −0.92; 95% CI: 1.30 to −0.55; p < 0.001) and jump performance (moderate effect: SMD = 0.62; 95% CI: 0.33 to 0.91; p < 0.001). However, FCT failed to significantly improve maximal strength (small effect: SMD = 0.43; 95% CI: 0.04 to 0.89; p = 0.07). Subgroup analyses revealed that control group type, training frequency, training load, and participant characteristics were moderating variables influencing training effects.

**Conclusion:**

French contrast training is more effective than traditional resistance training for enhancing lower limbs explosive performance in healthy adults. However, there is not enough evidence to show it is superior to traditional complex training or that it improves maximal strength. Future research should aim to optimize FCT protocols and explore long-term adaptability across different populations.

**Systematic Review Registration:**

https://www.crd.york.ac.uk/prospero/display_record.php, identifier CRD420251000409.

## Introduction

Complex Training (CT) is widely used to improve athletic performance, particularly for athletes. CT typically combines a high-load compound exercise (e.g., 85%1RM back squat) with a biomechanically similar high-velocity plyometric exercise (e.g., jump) in a single session, aiming to induce post-activation potentiation (PAP) and optimize force production (Dietz and Peterson, 2012). Previous studies have demonstrated the efficacy of CT in enhancing lower limb athletic performance. For example, Berriel et al. demonstrated that a 4-week CT program effectively improved jump height, it performed twice weekly and consisted of 3RM back squat followed by jump (Berriel et al., 2022); Liu et al. observed that CT significantly improved both jump height and maximal strength after a 12-week intervention, it performed twice weekly and consisted of 85%1RM back squat followed by jump and 85%1RM deadlift followed by 60-m sprints (Liu et al., 2022). A systematic review by Kasim et al. confirmed the efficacy of CT in improving lower limb jump height and maximal strength, and recommended implementing CT protocols over 6–12 weeks, with a training frequency of two–three sessions per week (Kasim et al., 2025). The effectiveness of CT is attributed to several mechanisms, including myosin light chain phosphorylation, increased calcium sensitivity, heightened neural drive, and greater recruitment of type II muscle fibers (Grgic, 2020; Xenofondos et al., 2010). Additionally, CT that alternates between high-load and self-weighted plyometrics exercises can effectively improve both ends of an athlete’s force velocity curve (F-V curve) (Stone et al., 2003). The F-V curve illustrates an inverse correlation between muscular force production and contraction velocity, wherein higher forces are produced at lower movement velocity, and lower forces at high velocity (Jiménez-Reyes et al., 2017). For example, when athletes perform a squat at their one repetition maximum (1RM), they generate a high level of force but at a slow movement velocity. In contrast, a countermovement jump (CMJ) involves high movement velocity but with a low load and minimal force. The F-V curve provides a detailed mechanical characterization of the relationship between muscle contraction velocity and load during resistance training (Lindberg et al., 2021). Thus, the F-V curve serves as a theoretical foundation for guiding athletes in developing personalized resistance training protocols (de Lacey et al., 2014). For example, athletes often perform high-load squats (≥85% 1RM) to increase maximal strength. They also frequently utilize light-to-moderate jump squats (30%–50% 1RM) to enhance speed-strength. Additionally, athletes incorporate plyometric exercises or assisted jumps to focus on maximizing their speed. However, traditional CT fails to consider the simulation of athletes with light-to-moderate load, potentially limiting its training benefits (Cormie et al., 2011).

French Contrast Training (FCT) is a novel training method designed to address limitations by considering training adaptations across the F-V curve (Long et al., 2023). It combines high-load, low-to moderate-load, and plyometric exercises in CT training. FCT consists of four sequential exercises phase (Long et al., 2023; Elbadry et al., 2019): 1) heavy compound exercise (e.g., 80%–90%1RM squat); 2) plyometric exercises (e.g., vertical jump); 3) light-to-moderate load compound exercise that maximizes movement velocity (e.g., ∼30% 1RM barbell weighted jumps); and 4) a plyometric exercise (often assisted). This structured protocol systematically stimulates adaptations across the F-V curve, aiming to optimize neuromuscular function (Dietz and Peterson, 2012; Kons et al., 2023). The philosophy of FCT aims to use four sequential exercise phases to elicit a physiological response in athletes and to train along the F-V curve. Despite its promising applications, current FCT studies are limited by small sample sizes and intervention protocol heterogeneity. For instance, Salam et al. found that third-weekly FCT with progressive loading significantly improved jump performance in athletes compared to routine training (Salam and Sherif, 2020). Rebelo et al. reported that twice-weekly FCT enhanced maximal strength in adolescents but did not significantly improve jump performance (Rebelo et al., 2023). Zhang et al. demonstrated that twice-weekly FCT significantly improves both jump performance and maximal strength in healthy adults compared to cluster training (Zhang, 2022). These inconsistencies may arise from factors such as participant characteristics, training loads, and the control group types.

Therefore, systematic review and meta-analysis are warranted to quantitatively synthesize the available evidence on FCT’s effectiveness in improving athletic performance. The objective of this meta-analysis was to assess the effects of FCT on jump, sprint, and maximal strength performance in healthy adults, while also exploring potential moderators such as control group type, training frequency, training load, and participant characteristics. We hypothesized that FCT would significantly enhance lower limb muscle strength and explosive performance in healthy adults compared to traditional training methods (e.g., CT, or resistance training).

## Methods

This systematic review and meta-analysis were conducted following the PRISMA guideline (Page et al., 2021). It was also registered with PROSPERO (CRD420251000409).

### Data sources, search strategy, and study selection

Two independent reviewers (K.Z. and Z.Z.) conducted a comprehensive literature search in five databases (PubMed, Web of Science, Embase, EBSCO, and CNKI) from inception to 25 February 2025. A detailed search strategy is provided in Supplementary Table S1. In addition, the reference lists of all relevant publications were manually screened to identify additional eligible studies.

Studies were included based on the following criteria: 1) Population: participants were healthy adults aged 18 years or older who had no neuromuscular or musculoskeletal disorders; 2) Intervention: the interventions focused on FCT; 3) Comparison: the control group utilized various training methods, including traditional complex training, resistance training, plyometrics, routine training or Cluster training; 4) Outcomes: the studies reported at least one of the following outcomes: jump, sprint or maximal strength performance; 5) Study design: all included studies employed a randomized controlled trail (RCT).

Studies were excluded if they were:1) animal trials; 2) case-control studies; 3) review articles; 4) conference articles; 5) repeated publications; and 6) acute experiments (≤1 week).

### Data extraction, outcome measures, and risk of bias assessment

Two independent reviewers (Z.Z. and Z.M.) extracted relevant data from included studies, including the authors, publication year, sample size, participant characteristics, exercise design, and outcome measures. Any disagreement between the two authors was discussed with K.Z. until a consensus was reached. The mean and standard deviation of each outcome in post-tests were extracted for each included study. If the post-test values were not available, they were calculated using the following formulas, where the correlation coefficient (Corr) was set at 0.5 (Chandler et al., 2019; Xue et al., 2019).

Meanpost=Meanpre+Meanchange

$$\text{SDpost}=\frac{2\times \text{Corr}\times \text{SDpre}+\sqrt{4\times {\text{Corr}}^{2}\times {\text{SDpre}}^{2}-4\times \left({\text{SDpre}}^{2}-{\text{SDchange}}^{2}\right)}}{2}$$

If the relevant outcome was unavailable, corresponding authors were contacted. In cases where the date could not be obtained, WebPlotDigitizer (version 4.6) was used to extract data from graphical representations (Petzold et al., 2023). Two independent reviewers (Z.Z. and Z.M.) repeated the graphic data extraction and cross-checked the data. Data were reextracted when there was >1% difference in the pairs (Drevon et al., 2017).

In the included studies, we categorized the outcome measures into three domains: 1) jump performance, assessed using CMJ, squat jump (SJ), and standing long jump (STJ); 2) sprint performance, evaluated through 30-m or 50-m sprint times; 3) maximal strength, determined by one-repetition maximum (1RM) tests, specifically the back squat (BS) and deadlift (DL).

Two investigators (K.Z. and Z.Z.) independently evaluated the risk of bias in the included studies using the Cochrane Collaboration’s risk of bias tool (Cump et al., 2019). Any disagreement between the two authors was discussed with Z.M. The assessment was based on the following criteria: 1) selection bias, 2) performance bias, 3) detection bias, 4) attrition bias, 5) reporting bias, and 6) other sources of bias. A study was classified as having a high risk of bias if one or more of these criteria indicated a high risk. Conversely, studies were categorized as having a low risk of bias if all criteria were rated as low. In cases where some criteria were rated as low and others as high, the studies were classified as having a moderate risk of bias.

### Statistical analysis and grading evidence

Effect size (ES) was calculated as standardized mean difference (SMD; Hedges’s g), with 95% confidence interval (CI). ES was classified as trivial (<0.2), small (0.2–0.49), moderate (0.5–0.79), or large (>0.8) (Cohen, 1988). Meta-analysis was performed in Stata v18.0 (STATA Corp., College Station, TX) using the inverse variance method. The heterogeneity was assessed by measuring the inconsistency (*I* ^*2*^ statistic) of intervention effects among the trials. The level of heterogeneity was interpreted according to guidelines from the Cochrane Collaboration: trivial (<25%), low (25–50%), moderate (50–75%), and high (>75%) (Higgins et al., 2003). A random-effects model was used to estimate the pooled effect in anticipation of heterogeneity across the studies due to differences in participants and intervention characteristics. The publication bias was assessed by the funnel plot and Egger’s test. Subgroup analysis was used to analyze the possible sources of heterogeneity. If a significant asymmetry was detected, we used the Trim and Fill method for sensitivity analysis of the results. All the statistical significance was set at p < 0.05.

Additionally, the quality of evidence for outcomes was evaluated using the Grading of Recommendations Assessment, Development and Evaluation (GRADE), which characterizes the evidence on the study limitations, imprecision, inconsistency, indirectness, and publication bias (Balshem et al., 2011; Meader et al., 2014).

## Results

**FIGURE 1:**
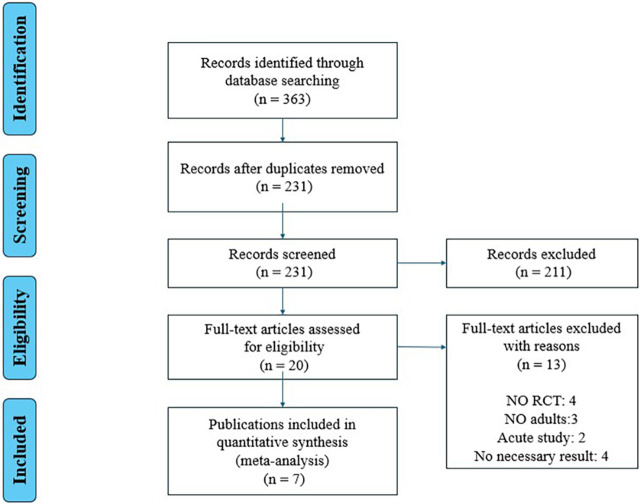
Study flowchart.

The flow diagram of screening is shown in Figure 1. A total of 363 relevant studies were retrieved (PubMed n = 52, Web of Science n = 82, Embase n = 48, EBSCO n = 152, CNKI n = 29), and 343 studies were excluded after reviewing the titles and abstracts. After the evaluation of full texts, 13 of the 20 studies were removed, and thus seven studies were included in the following analyses (Table 1).

**TABLE 1:** Characteristics of the included studies (N = 7).

Study	Participant characteristics	Study design	Sample size	Mean age	FCT programs	CON programs	Frequency/Period	Outcome measures
Meader et al. (2014)	Adult athletes	RCT	FCT:15 CON:15	21.0 ± 3.0	[1. BS (60%–85%1RM) Reps.6-10 2. CMJ Reps.6-10 3. Loaded CMJ Reps.6-10 4. Band assisted jump Reps.6-10] *Set.1-4	Routine training	3/week 12weeks	Sprint: 50 m sprint↑ Others: RHR→; VC↑; AP↑; Hand grip strength test↑
Salam and Sherif (2020)	Adult athletes	RCT	FCT:10 CON:10	FCT:21.1 ± 1.4 CON:21.0 ± 1.6	NR	Routine training	3/week 10weeks	Jump: STJ↑ Maximal Strength: Max legs dynamic strength↑ Others: BMD↑; STTJ↑ Specialized test↑
Noufal et al. (2024)	Adult athletes	RCT	FCT:15 CT:15 CON:15	19.4 ± 1.1	[1. BS (60%–85%1RM) Reps.4-6 2. Box jump Reps.6-10 3. Loaded CMJ (30%–40%1RM) Reps.4-6 4. Band assisted jump Reps.6-10] *Set.3-4	CT: [1. BS (60%–85%1RM) Reps.4-6 2. Box jump Reps.6-10] *Set.3-4 CON: Routine training	3/week 12weeks	Sprint: 50 m sprint↑ Others: RHR→; VC↑; VO_2_max↑; AP↑; ME↑; Illinois agility↑
Liu (2020)	Adult athletes	RCT	FCT:8 CON:8	FCT:19.3 ± 1.1 CON:18.6 ± 0.7	[1. BS (85%1RM)Reps.5 2. DJ Reps.5 3. Barbell DJ (30%1RM)Reps.5 4. Band assisted jump Reps.5] *Set.4	1. BS (85%1RM) Reps.5*Set.4 2. DJ Reps.5*Set.4 3. barbell DJ (30%1RM) Reps.5*Set.4 4. Band assisted jump Reps.5*Set.4	3/week 8weeks	Jump: STJ↑ Sprint: 30 m sprint↑ Maximal Strength: 1RM BS↑ Others: STTJ↑
Valappil et al. (2024)	Adult athletes	RCT	FCT:8 CON:8	FCT:22.0 ± 0.7 CON:21.7 ± 0.8	[1.BS (85%1RM)Reps.5 2. DJ Reps.4 3. Barbell DJ (30%1RM)Reps.4 4. Accelerated quadruple frog jump Reps.1] *Set.4	1. BS (85%1RM) Reps.5*Set.4 2. DJ Reps.4*Set.4 3. Barbell DJ (30%1RM) Reps.4*Set.4 4. Accelerated quadruple frog jump Reps.1*Set.4	2/week 8weeks	Jump: STJ↑; CMJ↑ Sprint: 30 m sprint↑ Maximal Strength: 1RM BS↑ Others: STTJ↑; Specialized test↑
Chen (2024)	Adult athletes	RCT	FCT:10 CON:10	FCT:18.7 ± 1.8 CON:19.2 ± 1.8	[1. Hex bar SJ (80%–85%1RM) Reps.2 2. DJ Reps.8 3. Weighted SJ (25%1RM)Reps.5 4. Band-assisted alternating split SJ Reps.8] *Set.4	1. Hex bar SJ (80%–85%1RM) Reps.8*Set.4 2. DJ Reps.8*Set.4 3. Weighted SJ (25%1RM) Reps.8*Set.4 4. Band-assisted alternating split SJ Reps.8*Set4	3/week 8weeks	Jump: STJ↑; CMJ↑; SJ↑ Sprint: 30 m sprint↑ Maximal Strength: 1RM BS↑; 1RM DL↑ Others: Specialized test↑; RSI↑
Zhang (2022)	Adult students	RCT	FCT:16 CLT:16 CON:16	FCT:22.1 ± 1.8 CLT:21.9 ± 1.7 CON:22.4 ± 1.6	[1. LR (80%1RM)Reps.5 2. Hurdle jump (20 cm)Reps.5 3. Dumbbell half SJ (10%1RM)Reps.5 4. Band assisted jump Reps.5] *Set.4	CLT: 1. LR (60%1RM) Reps.12+(70%1RM) Reps.8+(80%1RM) Reps.4 CON: Routine training	2/week 8weeks	Jump: CMJ↑; SJ↑ Maximal Strength: 1RM LR↑ Others: CMJ power↑; SJ power↑

1RM, One Repetition Maximum. 1RM BS, 1RM, Back Squat. 1RM DL, 1RM, deadlift. 1RM LR, 1RM, Leg Raise; AP, Anaerobic Power; BMD, Bone Mineral Density; BS, Back Squat; CLT, Cluster Training; CMJ, countermovement jump; CON, Control Group; CT, Complex Training; DJ, Drop Jump; FCT, French Contrast Training; LR, Leg Raise; ME, Muscular Endurance; NR, Not Reported. reps, Repetitions. RHR, Resting Heart Rate; RSI, Reactive Strength Index; SJ, Squat Jump; STJ, Standing Long Jump; STTJ, Standing Triple Jump; VC, vital capacity.

### Participant characteristics

A total of 195 healthy adults were included, comprising 62 females and 133 males, with a mean age ranging from 18.6 to 22.4 years. Of these, 147 participants were trained athletes in soccer (Salam and Sherif, 2020), field hockey (Noufal et al., 2024; Valappil et al., 2024), long jump (Liu, 2020), tennis (Chen, 2024), and kayaking (Ran, 2023), and 48 were untrained.

### Training protocol

There were variations in the exercises included in the FCT structured program.

1. Heavy compound exercise phase: four studies (Noufal et al., 2024; Valappil et al., 2024; Liu, 2020; Chen, 2024) utilized barbell back squats. Among them, two studies (Noufal et al., 2024; Valappil et al., 2024) implemented progressive load (60%–85%1RM over 12 weeks), while two studies (Liu, 2020; Chen, 2024) used a fixed load of 85% 1RM. One study (Zhang, 2022) employed leg press at 80% 1RM, and another (Ran, 2023) used trap bar deadlifts at 80%–85% 1RM.
2. Plyometric exercises phase: three studies (Liu, 2020; Chen, 2024; Ran, 2023) used bodyweight depth jumps; others used progressive box jumps (Valappil et al., 2024),hurdle jump (Zhang, 2022) and reverse lunge jumps (Noufal et al., 2024).
3. Light-to-moderate load compound exercise that maximizes movement velocity phase: four studies (Valappil et al., 2024; Liu, 2020; Chen, 2024; Ran, 2023) adopted barbell jump squats, with either fixed (30%1RM) (Liu, 2020; Chen, 2024) or progressive loads (30%–50%1RM) (Valappil et al., 2024; Ran, 2023). One study (Zhang, 2022) applied dumbbell jump squats at 10% 1RM, while another (Noufal et al., 2024) utilized loaded (unreported load) reverse lunge jumps.
4. Assisted or accelerated plyometric exercise phase: four studies (Zhang, 2022; Noufal et al., 2024; Valappil et al., 2024; Chen, 2024) used resistance band-assisted vertical jumps. One study (Ran, 2023) implemented band-assisted split jumps, while another (Liu, 2020) focused on accelerating frog jumps.

Among the included studies, training durations ranged from 8 to 12 weeks. Three studies (Liu, 2020; Chen, 2024; Ran, 2023) implemented 8-week protocol, with training frequencies of two (Liu, 2020) or three sessions (Chen, 2024; Ran, 2023) per week. Two studies (Salam and Sherif, 2020; Zhang, 2022) applied 10-week interventions with frequencies of two (Zhang, 2022) or three (Salam and Sherif, 2020) sessions per week. While another two studies (Noufal et al.; Valappil et al., 2024) employed 12-week protocols with thrice-weekly sessions.

Control group protocols varied across studies. Specifically, three studies (Salam and Sherif, 2020; Noufal et al., 2024; Valappil et al., 2024) used routine training, with one (Valappil et al., 2024) including complex training (CT); four studies (Zhang, 2022; Liu, 2020; Chen, 2024; Ran, 2023), used equal-load training (ELT). Equal-load training was a standalone method that utilized exercises with loads that correspond to FCT. For example, an athlete completes four sets of 85% 1RM barbell squats followed by four sets of jumping exercises, then four sets of 30% 1RM barbell weighted squat jumps, and finally four sets of elastic band-assisted jumps. Each exercise phase must be completed independently with 5–10 min of rest per phase. Additionally, one study (Zhang, 2022) included a Cluster training group. Cluster training was the method of adding “rest intervals between reps” to each set of a heavy weight training program (>80% 1RM). For example, in a set of five repetitions of 5RM barbell squats, add a 15–30 s “repetition interval” between each repetition, and then add a 2–5 min “set interval” between each set.

### Outcome measurements

#### Jump performance

Five studies (Salam and Sherif, 2020; Zhang, 2022; Liu, 2020; Chen, 2024; Ran, 2023) assessed jump performance. Among them, four studies (Salam and Sherif, 2020; Liu, 2020; Chen, 2024; Ran, 2023) measured STJ using a tape measure, and three (Salam and Sherif, 2020; Liu, 2020; Chen, 2024) of them also included the standing triple jump. Four studies (Zhang, 2022; Liu, 2020; Chen, 2024; Ran, 2023) reported CMJ. Ran et al. (Ran, 2023) used a contact mat, and Zhang et al. (Zhang, 2022) employed the Jump wireless system. Two studies (Liu, 2020; Chen, 2024)applied a force platform.

#### Sprint performance

Five studies (Noufal et al., 2024; Valappil et al., 2024; Liu, 2020; Chen, 2024; Ran, 2023) assessed sprint ability. Including 30-m (Liu, 2020; Chen, 2024; Ran, 2023) and 50-m (Noufal et al. (2024); Valappil et al., 2024) sprint tests measured with stopwatches and Brower Timing System (Ran, 2023).

#### Maximal strength

Five studies (Salam and Sherif, 2020; Zhang, 2022; Liu, 2020; Chen, 2024; Ran, 2023) reported maximal strength. Among them, three studies (Liu, 2020; Chen, 2024; Ran, 2023) used 1RM BS. Ran et al. (Ran, 2023) additionally measured 1RM DL and analyzed peak bar velocity using the Gymaware system. One study (Zhang, 2022) estimated 1RM leg press based on a 10RM test using the O’Conner formula. One study (Salam and Sherif, 2020) measured maximal leg strength but did not indicate the method.

#### Other outcomes

Including studies that also measured outcomes such as agility, physiological indicators, and specific motor skills. One study (Valappil et al., 2024) assessed agility using the Illinois Agility Test. Three studies (Salam and Sherif, 2020; Noufal et al., 2024; Valappil et al., 2024) reported the physiological and biochemical parameters, including anaerobic power (Margaria–Kalamen stair sprint test), resting heart rate (spirometry), vital capacity (Queen’s College Step Test), and estimated VO_2_max. One study used dual-energy X-ray absorptiometry to evaluate bone mineral density (Salam and Sherif, 2020). Three studies (Salam and Sherif, 2020; Liu, 2020; Ran, 2023) assessed sport-specific skills, including football movement combinations, long jump technique, and rowing performance using a rowing ergometer. Reactive strength index was evaluated in one study (Ran, 2023) using drop jumps on a contact mat, calculated as the ratio of jump height to ground contact time.

#### Risk of bias

**FIGURE 2:**
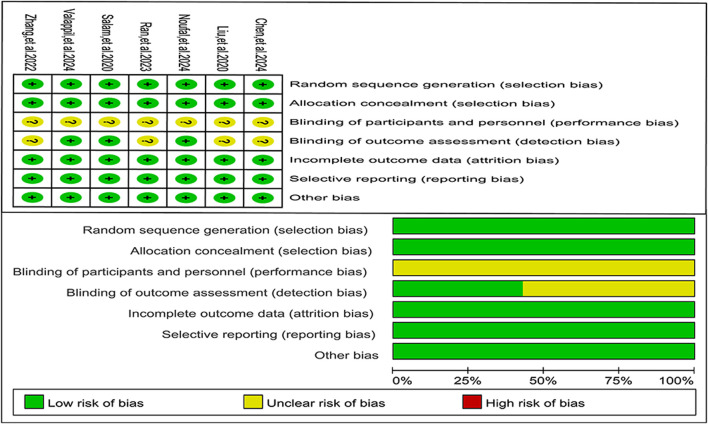
Risk of bias in the included studies.

The results of the quality assessment for the seven included studies were presented in Figure 2. All studies exhibited a moderate risk of performance bias, primarily because none of them explicitly reported the type or method of blinding used during the interventions.

#### Meta-analysis

We conducted subgroup analyses of control group type (e.g., FCT vs CT), participant characteristics (trained vs untrained), load (progressive vs fixed load), and training frequency (twice vs thrice per week) to identify potential moderators of training effects (Table 2).

**TABLE 2:** Subgroup analysis results regarding the effects of FCT on Lower Limb Performance.

Outcomes	Variables	No. of participants	SMD (95%CI)	*P value*	Test of heterogeneity	
					*P value*	*I* ^*2*^ (%)
Jump	Control Group					
	Routine training	42	0.86 (0.35,1.37)	*0.001*	*0.16*	*19.32*
	ELT	54	0.61 (0.17,1.06)	*0.01*	*0.18*	*20.29*
	Cluster training	32	0.34 (-0.15,0.84)	*0.17*	*0.94*	*0*
	Training Frequency					
	Twice/week	80	0.71 (0.34,1.08)	*0.001*	*0.17*	*21.79*
	Thrice/week	48	0.48 (0.05,0.91)	*0.03*	*0.26*	*4.81*
	Training Load					
	Fixed	88	0.78 (0.41,1.16)	*0.001*	*0.14*	*27.18*
	Progressive	40	0.32 (-0.13,0.76)	*0.16*	*0.7*	*0*
	Participant Characteristics					
	Trained	64	0.53 (0.13,0.93)	*0.01*	*0.21*	*16.66*
	Untrained	64	0.72 (0.33,1.11)	*0.001*	*0.20*	*14.47*
Sprint	Control Group					
	Routine training	30	−1.42 (-1.99, −0.84)	*0.001*	*0.99*	*0*
	ELT	26	−0.77 (-1.34, −0.19)	*0.01*	*0.48*	*0*
	Complex training	15	−0.38 (-1.10,0.34)	*0.31*	-	-
	Training Frequency					
	Twice/week	8	−0.33 (-1.32,0.66)	*0.51*	-	-
	Thrice/week	63	−1.01 (-1.39, −0.55)	*0.001*	*0.27*	*3.17*
	Training Load					
	Fixed	16	−0.73 (-1.47,0)	*0.05*	*0.23*	*0*
	Progressive	55	−0.98 (-1.44, −0.53)	*0.001*	*0.17*	*19.65*
Maximal Strength	Control Group					
	Routine training	26	1.41 (0.79,2.03)	*0.001*	*0.81*	*0*
	ELT	36	0.01 (-0.46,0.47)	*0.97*	*0.96*	*0*
	Cluster training	16	0.13 (-0.56,0.83)	*0.71*	-	-
	Training Frequency					
	Twice/week	40	0.47 (-0.26,1.20)	*0.21*	*0.02*	*58.81*
	Thrice/week	38	0.38 (-0.20,0.97)	*0.2*	*0.10*	*36.7*
	Training Load					
	Fixed	48	0.41 (-0.18,0.99)	*0.17*	*0.05*	*47.97*
	Progressive	30	0.46 (-0.29,1.22)	*0.23*	*0.05*	*50.79*
	Participant Characteristics					
	Trained	46	0.28 (-0.23,0.78)	*0.29*	*0.12*	*30.75*
	Untrained	32	0.72 (-0.12,1.56)	*0.09*	*0.02*	*61.64*

ELT, Equal-Load Training.

### Effect of FCT on jump performance

Five publications (Salam and Sherif, 2020; Zhang, 2022; Liu, 2020; Chen, 2024; Ran, 2023) showed that FCT could significantly improve jump height as compared to control groups.

**FIGURE 3:**
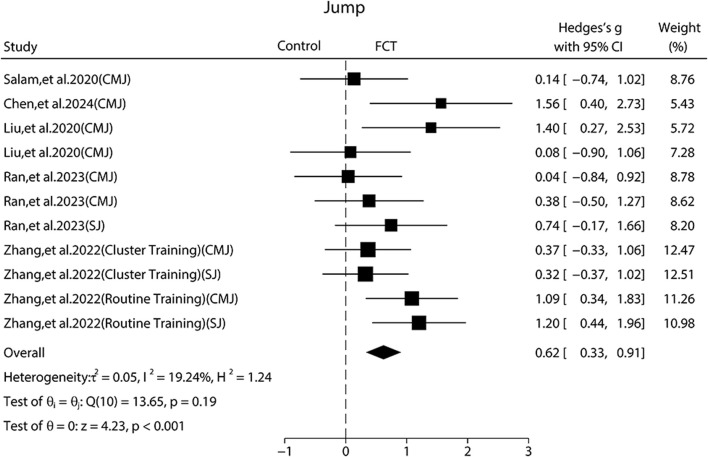
Forest plot of the effects of FCT on jump. CMJ: Countermovement Jump; SJ: Squat Jump.

The pooled ES of jump performance was moderate and significant (SMD_pooled_ = 0.62, 95% CI 0.33 to 0.91, p < 0.001, Figure 3) with low heterogeneity (*I* ^*2*^ = 19.24%, p = 0.19). The funnel plot (Figure 6a) and Egger’s test (p = 0.419) indicated no publication bias.

Subgroup analyses revealed that jump type contributed significantly to the effects of jump performance. Specifically, the ES in routine training was significant and large (SMD = 0.86, 95% CI 0.35 to 1.37, p < 0.001) and moderate in the ELT group (SMD = 0.61, 95% CI 0.17 to 1.06, p = 0.01) but not significantly in cluster training (SMD = 0.34, 95% CI 0.15 to 0.84, p = 0.17). For participant characteristics, the ES was moderate and significant in untrained (SMD = 0.72, 95% CI 0.33 to 1.11, p < 0.001) and trained (SMD = 0.53, 95% CI 0.13 to 0.93, p = 0.01). Regarding the load, fixed load produced a moderate and significant ES (SMD = 0.78, 95% CI 0.41 to 1.16, p < 0.001), while the progressive load produced a small and not significant ES (SMD = 0.32, 95% CI –0.13 to 0.76, p = 0.70). Training frequency also influenced the outcomes. The ES was moderate and significant twice per week (SMD = 0.71, 95% CI 0.34 to 1.08, p < 0.001) and small in thrice per week (SMD = 0.48, 95% CI 0.05 to 0.91, p = 0.03).

### Effect of FCT on sprint performance

Five publications (Noufal et al.; Valappil et al., 2024; Liu, 2020; Chen, 2024; Ran, 2023) showed that FCT could significantly improve sprint as compared to control groups.

**FIGURE 4:**
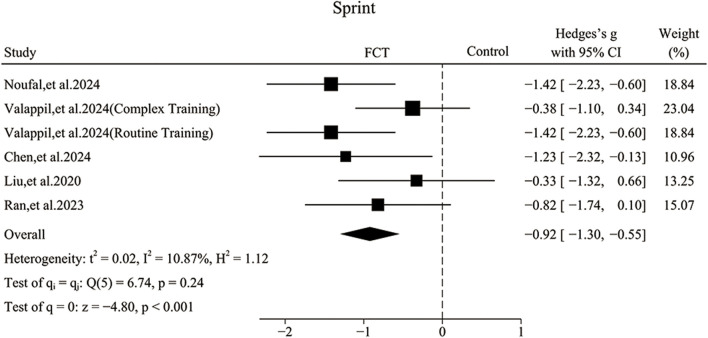
Forest plot of the effects of FCT on sprint.

The pooled ES of sprint was large and significant (SMD_pooled_ = −0.92, 95% CI = −1.30 to −0.55, p < 0.001, Figure 4) with low heterogeneity (*I* ^*2*^ = 10.87%, p = 0.24). The funnel plot (Figure 6b) and Egger’s test (p = 0.844) indicated no publication bias.

Subgroup analyses revealed that control group type contributed significantly to the effects of sprint. Specifically, the ES was large and significant in routine training (SMD = −1.42, 95% CI –1.99 to −0.84, p < 0.001) and moderate in ELT group (SMD = −0.77, 95% CI –1.34 to −0.19, p = 0.01), while it was small and not significant in complex training (SMD = −0.38, 95% CI –1.10 to 0.34, p = 0.31). Regarding the load, the ES was large and significant in progressive load (SMD = −0.98, 95% CI –1.44 to −0.53, p < 0.001) and moderate in fixed load (SMD = −0.73, 95% CI –1.47 to 0.00, p = 0.05). For training frequency, thrice per week produced a large and significant ES (SMD = −1.01, 95% CI –1.39 to −0.62, p < 0.001), while twice per week produced a small and not significant ES (SMD = −0.33, 95% CI –1.32 to 0.66, p = 0.51).

### Effect of FCT on maximal strength

Three publications (Salam and Sherif, 2020; Zhang, 2022; Chen, 2024) showed that FCT can significantly improve maximal strength as compared to control groups, while two publications (Liu, 2020; Ran, 2023) reported that FCT cannot significantly enhance maximal strength compared to control groups.

**FIGURE 5:**
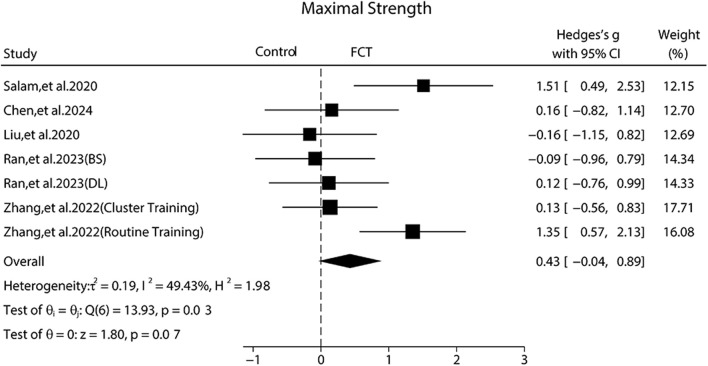
Forest plot of the effects of FCT on maximal strength. BS: Back Squat; DL: Deadlift.

**FIGURE 6:**
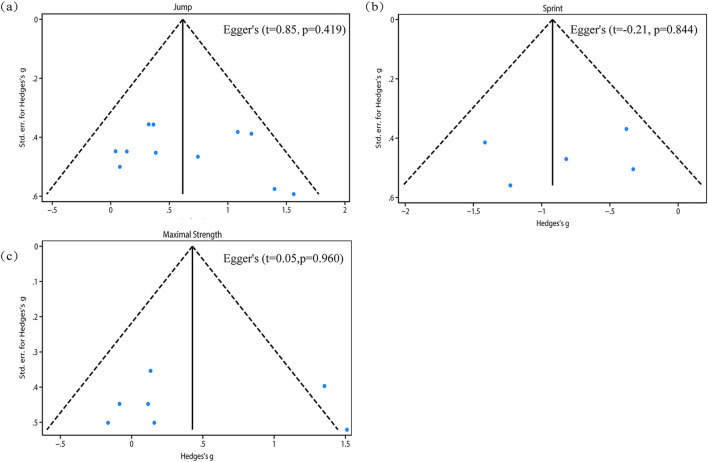
Publication bias funnel: **(a)** jump; **(b)** sprint; **(c)** maximal strength.

The pooled ES of maximal strength was small and not significant (SMD_pooled_ = 0.43, 95% CI –0.04 to 0.89, p = 0.07, Figure 5), with moderate heterogeneity (*I* ^*2*^ = 49.43%, p = 0.03). The funnel plot (Figure 6c) and Egger’s test (p = 0.960) indicated no publication bias.

Subgroup analyses showed that control group type contributed significantly to the effects of maximal strength. Specifically, the ES was large and significant in routine training (SMD = 1.41, 95% CI 0.79 to 2.03, p < 0.001), while it was trivial and not significant in cluster training (SMD = 0.12, 95% CI –0.56 to 0.83, p = 0.71) and ELT group (SMD = 0.01, 95% CI –0.46 to 0.47, p = 0.97). For participant characteristics, the ES was moderate and not significant in untrained (SMD = 0.72, 95% CI –0.12 to 1.56,p = 0.09) and small in trained (SMD = 0.28, 95% CI –0.23 to 0.78,p = 0.29). Regarding the load, both the progressive (SMD = 0.46, 95% CI –0.29 to 1.22,p = 0.23) and fixed load (SMD = 0.41, 95% CI –0.18 to 0.99, p = 0.17) produced small and not significant ES. Similarly, in terms of training frequency, twice per week (SMD = 0.47,95% CI –0.26 to 1.20, p = 0.21) or thrice per week (SMD = 0.38, 95% CI –0.20 to 0.97, p = 0.20) produced small and not significant ES.

### GRADE assessment

The quality of evidence for each outcome was rated as moderate based on the GRADE criteria. Detailed evaluations using the GRADE framework, including assessments of risk of bias, inconsistency, imprecision, indirectness, and publication bias (Supplementary Table S2).

## Discussion

These findings highlight that FCT is an effective method for enhancing jump and sprint performance in healthy adults, but it did not show a significant advantage for maximal strength gains in the lower limbs compared to traditional training (e.g., CT and resistance training). Control group type, training frequency, training load, and participant characteristics are important moderating variables that influence the effectiveness of FCT.

FCT offers the benefit of enhancing explosive power in the lower body more effectively than traditional training methods. There are several possible explanations for this result. First, FCT provides comprehensive neuromuscular stimulation across the full spectrum of the F-V curve through its strategically structured exercise sequence (Cormier et al., 2022), significantly enhancing explosive performance. Specifically, heavy compound exercises increase maximal force output by enhancing neural drive and recruiting high-threshold motor units, thus shifting the curve upwards in the high force, low velocity domain (Rong et al., 2025). Plyometric and light-to-moderate load compound exercise target the moderate and high velocity domain, facilitating improvements in the rate of force development (RFD), motor unit firing rates, and muscle tendon stiffness (Maffiuletti et al., 2016; Ramírez-delaCruz et al., 2022). Furthermore, assisted plyometric exercises extend neuromuscular adaptations towards the extreme velocity end of the F-V curve by decreasing effective bodyweight load, allowing for muscle contractions at velocity beyond typical capabilities (Stien et al., 2020). This sequential and integrative method actively promotes an upward and rightward shift of the F-V curve, resulting in comprehensive improvements in explosive neuromuscular performance. This aligns with previous findings by Jiménez et al., who emphasized that training interventions encompassing the full spectrum of the F-V curve are more effective in enhancing explosive performance. Second, FCT enhances explosive strength through central and peripheral neuromuscular adaptations associated with the post-activation performance enhancement (PAPE) effect. PAPE refers to a short-term increase in muscle output following high intensity pre-loading (Uysal and Alexe, 2024). At the central level, it enhances α-motor neuron excitability, increases corticomotor output, and improves motor unit recruitment and synchronization (Hodgson et al., 2005). These changes help generate faster and more coordinated neural activation during explosive training (Elbadry et al., 2019). Xu et al. found that PAPE improves the activation of high-threshold motor units, which supports rapid force production (XU et al., 2025). At the peripheral level, stretch-shortening cycle (SSC) movements improve elastic energy use and increase muscle-tendon stiffness (Santos et al., 2024). Schoenfeld et al. demonstrated that repeated exposure to high loads across the F-V curve promotes the recruitment and hypertrophy of type II muscle fiber (Schoenfeld, 2010). Given these advantages, FCT may be effectively applied during pre-season or targeted explosive-strength training to improve jump performance in athletes from volleyball, basketball, or jumping disciplines. Cengizel et al. reported that FCT enhanced athletic performance as a pre-season activation strategy in basketball (Cengizel et al., 2025). However, future studies should further investigate the long-term effects of FCT on athletic performance.

In contrast, FCT demonstrated limited effects on maximal strength development. A potential reason is that strength gains typically require sustained mechanical tension, high training volume, and extended time under tension (TUT) (Bernárdez-V et al., 2022; Burd et al., 2012), conditions that are often not met in FCT protocols. Schoenfeld et al. emphasized that lifting at intensities above 85% of 1RM for multiple sets is essential for optimizing strength adaptations (Schoenfeld et al., 2021). However, FCT prioritizes movement velocity and neural potentiation over prolonged loading, which may reduce total mechanical strain. High-frequency explosive efforts in FCT may induce neuromuscular fatigue that compromises motor unit recruitment efficiency for maximal strength (Hung et al., 2025). Consequently, FCT may be better positioned as a specialized method for enhancing explosive performance rather than as a comprehensive strategy for maximal strength development. To address this limitation, we suggest integrating FCT with traditional resistance training in a periodized model. Alternating high-load sessions with FCT-based explosive training may allow athletes to benefit from both mechanical and neural stimuli, thereby supporting concurrent improvements in maximal strength and power. Additionally, the moderate heterogeneity was observed in maximal strength (*I* ^2^ = 49.43%). Several factors may have contributed to this heterogeneity, including the type of control group, training frequency, training load, and participant characteristics. These methodological differences likely diminished the comparability of the findings across studies. Future research should aim to use standardized training and testing protocols to reduce variations in strength outcomes.

Subgroup analyses revealed that FCT outcomes are influenced by key moderate variables, including control group type, training frequency, training load, and participant characteristics. Specifically, FCT is more effective than traditional training methods (e.g., resistance training) at improving lower limb explosive power in healthy adults. This is primarily due to plyometric exercises in FCT are better for improving explosive lower body performance than traditional resistance training, due to their training loads and patterns. However, extra plyometric exercises or light-to-moderate load compound exercises did not enhance maximal strength gains in FCT. This is because increases in maximal strength relate to high-load exercises (e.g., ≧85%1RM), while light-to-moderate load and plyometric exercises may provide ineffective stimuli (Schoenfeld et al., 2021). Another important finding is that the current evidence is not sufficient to confirm that FCT enhances lower limb explosive power more effectively than traditional CT. Valappil et al. (2024) proposed that FCT might be more effective than CT (29). They argued that FCT led to greater improvements in athletic performance (specifically in speed, agility, muscular endurance, and anaerobic strength) in field hockey players compared to CT. However, the difference in outcomes between the two groups was not statistically significant (p > 0.05). The potential benefits of FCT may be related to session exercise sequences rather than training load, as FCT is significantly more effective than ELT in enhancing lower limb explosive power. A larger sample size may be necessary to validate these findings. Additionally, data on biochemistry and hematology should be collected for further insights. A training frequency of twice weekly yielded optimal jump performance, while higher frequencies showed no additional benefit and may induce fatigue, particularly in untrained participants. In contrast, sprint performance appeared to benefit from more frequent exposures, possibly due to enhanced neuromuscular stimulation. This inconsistency may be due to participant characteristics or control group settings. The training frequency should be based on the athlete’s training status and recovery. One interesting finding is that fixed load protocols produced greater improvements in jump performance, likely due to more consistent high intensity exposure, whereas progressive loading may be better suited for long term strength development through gradual overload. Regarding participant characteristics, untrained participants seem to benefit more from FCT than trained individuals, as athletes experience a “ceiling effect” that limits their advantages. These findings highlight the importance of tailoring FCT protocols to individual training backgrounds and goals. Future research should further examine the effects of the aforementioned moderating variables on FCT to determine its actual value in athletic training practice.

### Limitations

Several limitations should be acknowledged. First, the total number of studies included in the analysis is small (n = 7), and many of these trials had limited sample sizes, often comprising fewer than 20 participants. This limitation reduces the generalizability of the findings and increases the risk of a type II error. Second, the FCT protocols used across studies varied considerably, particularly regarding exercise selection, training load, and frequency, introducing heterogeneity that may obscure accurate effect sizes. Third, none of the included trials implemented participant blinding, and outcome assessors were rarely blinded, increasing the risk of performance and detection bias. Additionally, the participant characteristics varied widely, ranging from recreationally active individuals to competitive athletes across various sports (e.g., track and field, basketball, soccer). Few studies conducted subgroup analyses to explore potential differences in responses among these diverse participants.

Future research should conduct randomized controlled trials with larger sample sizes to strengthen findings on FCT. Utilizing standardized FCT protocols is essential to reduce variability in exercise selection, training loads, and frequency, allowing for more accurate comparisons and effect size estimates. Additionally, future research should examine subgroups based on participant characteristics, such as training status or female athletes, to enhance understanding of individual responses to FCT.

## Conclusion

This review indicates that French Contrast Training (FCT) demonstrates superior efficacy in enhancing lower-limb explosive performance compared to traditional resistance training in healthy adults. However, current evidence remains insufficient to establish FCT as an optimized training method when compared to traditional complex training (CT). Furthermore, existing studies suggest that FCT may not confer significant additional benefits over traditional resistance or complex training in the development of maximal strength. These findings highlight both the potential and the limitations of FCT in athletic performance enhancement.

## Data Availability

The original contributions presented in the study are included in the article/Supplementary Material, further inquiries can be directed to the corresponding author.
