# Supplementary material for: The effects of French contrast training on lower limb athletic performance in healthy adults: a systematic review and meta-analysis
Source: Front Physiol. 2025 Aug 21;16:1672353. doi: 10.3389/fphys.2025.1672353 (PMC12408690; doi:10.3389/fphys.2025.1672353)
Supplement: Supplementary file 1 [file DataSheet1.docx]

Supplementary Material

**The Effects of French Contrast Training on Lower Limb Athletic Performance in Healthy Adults: A Systematic Review and Meta-Analysis**

**Ziren Zhao**^1, †^**, Zhili Ma**^2, †^**, Chao Wu^3^, Xin Zheng^1^, Tingyuan Liu^1^, Nijiao Deng^1^, Kaixiang Zhou^1*^**

^1^ College of Physical Education and Health Science, Chongqing Normal University, Chongqing, China;

^2^ Physical Education Department, University of International Business and Economics, Beijing, China;

^3^ Physical Education Department, University of International Business and Economics, Beijing, China;

*** Correspondence:**Kaixiang Zhou, PhD, College of Physical Education and Health Science, Chongqing Normal University, University Town, Shapingba District, Chongqing, China; e-mail address: [20230102@cqnu.edu.cn](mailto:20230102@cqnu.edu.cn).

**^†^ Equal contribution and first authorship:** These authors contributed equally to this work and share first authorship

**1 Supplementary Tables**

1.1 Table S1 Search Strategy.

1.2 Table S2 The quality of the evidence (GRADE).

1.3 Table S3 Data Sheet

**2 Supplementary Figure**

2.1 Figure S1 Leave-one-out sensitivity analysis plot

**Table S1 Search Strategy**

| Data | Keywords | Result |
| --- | --- | --- |
| Pubmed | ((French contrast training OR French contrast methods OR FCT OR FCM OR Contrast training OR Complex training) AND (Exercise performance OR Physical performance OR Explosive strength OR Jump performance)) | 52 |
| Web of Science | TS= ("French contrast training" OR "French contrast methods" OR "FCT" OR "FCM" OR "Contrast training" OR "Complex training") AND TS= ("Exercise performance" OR "Physical performance" OR "Explosive strength" OR "Jump performance") | 82 |
| Embase | (french contrast training OR french contrast methods OR fct OR fcm OR contrast training OR complex training) in Title Abstract Keyword AND (exercise performance OR physical performance OR explosive strength OR jump performance) in Title Abstract Keyword | 48 |
| EBSCO | ("French contrast training" OR "French contrast methods" OR "FCT" OR "FCM" OR "Contrast training" OR "Complex training") AND ("Exercise performance" OR "Physical performance" OR "Explosive strength" OR "Jump performance") | 152 |
| CNKI | ("French contrast training" OR "French contrast methods" OR "FCT" OR "FCM" OR "Contrast training" OR "Complex training") AND ("Exercise performance" OR "Physical performance" OR "Explosive strength" OR "Jump performance") | 29 |

Data ranges for all databases: From inception to February 25, 2025.

**Table S2 The quality of the evidence (GRADE)**

| **Quality assessment** | | | | | | | **No of participants** | | **Effect** | **Quality** | **Importance** |
| --- | --- | --- | --- | --- | --- | --- | --- | --- | --- | --- | --- |
| **No of experiments** | **Design** | **Risk of bias** | **Inconsistency** | **Indirectness** | **Imprecision** | **Other considerations** | **FCT** | **Control** | **Absolute** |  |  |
| **Jump Performance (Better indicated by lower values)** | | | | | | | | | | | |
| 5 | randomised trials | Serious^1^ | no serious inconsistency | no serious indirectness | no serious imprecision | none | 128 | 128 | SMD 0.62 higher (0.36 to 0.87 higher) | ⊕⊕⊕⊝ | CRITICAL |
|  |  |  |  |  |  |  |  |  |  | MODERATE |  |
| **Sprint Performance (Better indicated by lower values)** | | | | | | | | | | | |
| 5 | randomised trials | Serious^1^ | no serious inconsistency | no serious indirectness | no serious imprecision | none | 78 | 78 | SMD -0.92 higher (  -0.57 to  -1.27 higher) | ⊕⊕⊕⊝ | CRITICAL |
|  |  |  |  |  |  |  |  |  |  | MODERATE |  |
| **Maximal Strength (Better indicated by lower values)** | | | | | | | | | | | |
| 5 | randomised trials | Serious^1^ | no serious inconsistency | no serious indirectness | no serious imprecision | none | 71 | 71 | SMD 0.43 higher (0.1 lower to 0.75 higher) | ⊕⊕⊕⊝ | CRITICAL |
|  |  |  |  |  |  |  |  |  |  | MODERATE |  |

^1^ Risk of selection bias: The lack of building.

**Table S3 Data Sheet**

| Author Year | Variable | FCT Mean | FCT SD | Control  Mean | Control  SD | Sample  size |
| --- | --- | --- | --- | --- | --- | --- |
| Naufal et al.2024 | 50m sprint time (s) | 6.97 | 0.18 | 7.33 | 0.3 | 15 |
| Salam et al.2020 | Standing long jump (m) | 2.41 | 0.68 | 2.32 | 0.57 | 10 |
| Salam et al.2020 | Legs dynamic strength(kg) | 95.7 | 3.67 | 90.05 | 3.49 | 10 |
| Valappil et al.2024(Complex Training) | 50m sprint time (s) | 6.97 | 0.18 | 7.04 | 0.18 | 15 |
| Valappil et al.2024(Routine Training) | 50m sprint time (s) | 6.97 | 0.18 | 7.33 | 0.3 | 15 |
| Chen et al.2024 | Standing long jump (m) | 2.87 | 0.14 | 2.63 | 0.15 | 8 |
| Chen et al.2024 | Back squat(kg) | 132.5 | 13.36 | 130.63 | 8.21 | 8 |
| Chen et al.2024 | 30m sprint time (s) | 3.95 | 0.1 | 4.08 | 0.1 | 8 |
| Liu et al.2020 | Standing long jump (m) | 2.5 | 0.05 | 2.41 | 0.07 | 8 |
| Liu et al.2020 | Countermovement jump (m) | 0.52 | 0.1 | 0.51 | 0.14 | 8 |
| Liu et al.2020 | Back squat(kg) | 119.65 | 14.72 | 122 | 12.12 | 8 |
| Liu et al.2020 | 30m sprint time (s) | 4.34 | 0.2 | 4.45 | 0.4 | 8 |
| Ran et al.2023 | Standing long jump (m) | 2.51 | 0.23 | 2.5 | 0.23 | 10 |
| Ran et al.2023 | Countermovement jump (cm) | 44.46 | 4.57 | 42.41 | 5.6 | 10 |
| Ran et al.2023 | Squat jump (cm) | 41.55 | 5.58 | 37.22 | 5.56 | 10 |
| Ran et al.2023 | Back squat(kg) | 122 | 33.7 | 125 | 33.8 | 10 |
| Ran et al.2023 | Deadlift (kg) | 127.5 | 27.41 | 124 | 30.25 | 10 |
| Ran et al.2023 | 30m sprint time (s) | 4.66 | 0.17 | 4.89 | 0.34 | 10 |
| Zhang et al.2022(Cluster Training) | Countermovement jump (cm) | 26.5 | 3.8 | 25 | 4.2 | 16 |
| Zhang et al.2022(Cluster Training) | Squat jump (cm) | 26.4 | 3.2 | 25.4 | 2.8 | 16 |
| Zhang et al.2022(Routine Training) | Countermovement jump (cm) | 26.5 | 3.8 | 22.1 | 4.1 | 16 |
| Zhang et al.2022(Routine Training) | Squat jump (cm) | 26.4 | 3.2 | 22 | 3.9 | 16 |
| Zhang et al.2022(Cluster Training) | Leg raise (kg) | 146.51 | 35.18 | 141.43 | 39.06 | 16 |
| Zhanget al.2022(Routine Training) | Leg raise (kg) | 146.51 | 35.18 | 102.27 | 28.2 | 16 |


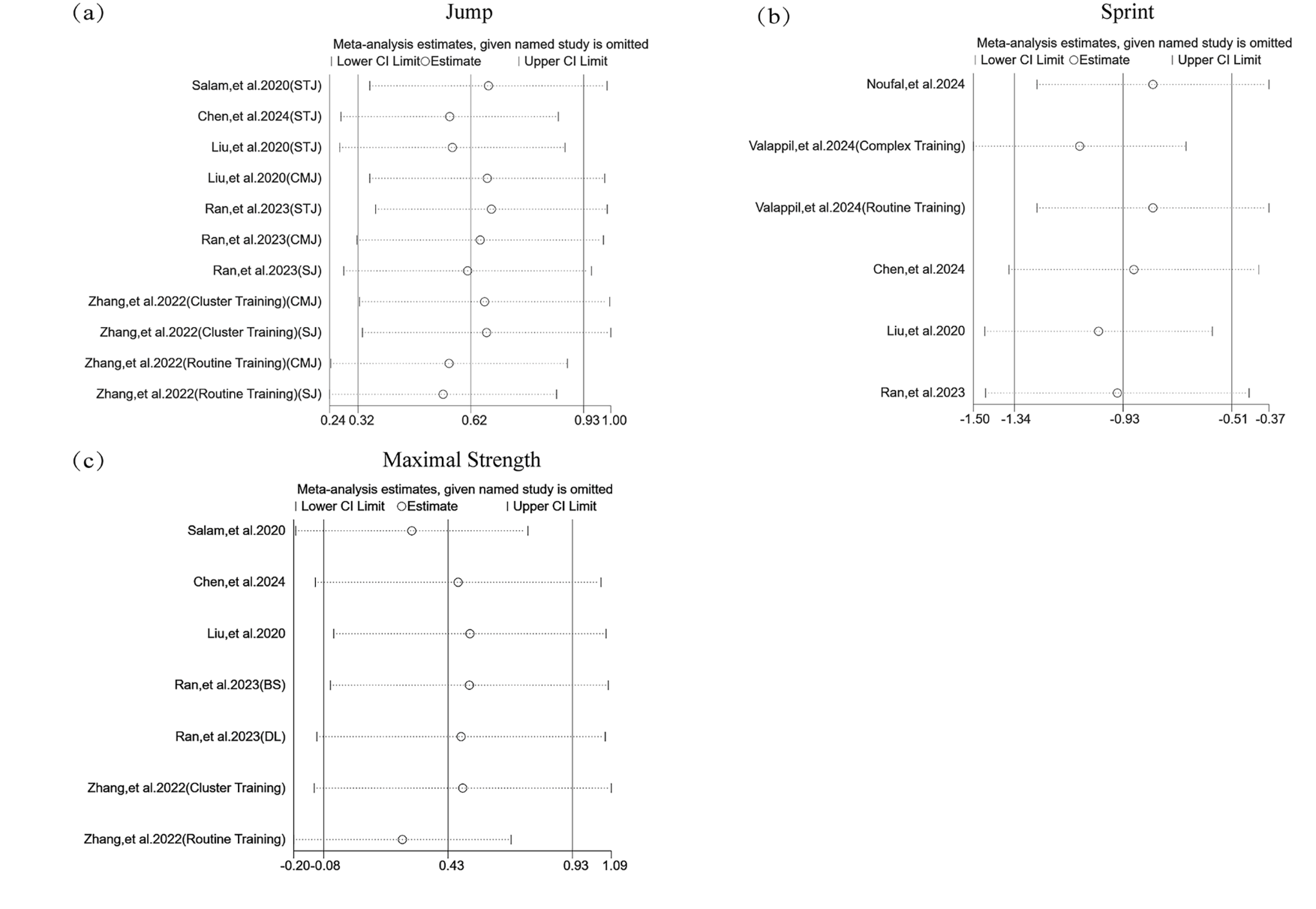


Figure S1 Leave-one-out sensitivity analysis plot.

**BS**, Back Squat. **CMJ**, Countermovement Jump. **DL**, Deadlift. **SJ**, Squat Jump. **STJ**, Standing Long Jump**.**
